# Retraction: Screening of potential biomarkers for polycystic ovary syndrome and identification of expression and immune characteristics

**DOI:** 10.1371/journal.pone.0330094

**Published:** 2025-08-12

**Authors:** The PLOS One Editors

Following the publication of this article [1], the corresponding author contacted PLOS requesting the retraction of the article, stating that the underlying code required for the bioinformatics analyses reported in this study, including the RFE algorithm, LASSO regression, and ssGSEA scripts, are no longer available. The corresponding author states that in the absence of the code, the validity of key methodological steps, such as the biomarker screening process (e.g., JDP2 and HMOX1) and the accuracy of the immune infiltration analysis cannot be verified.

In light of these concerns, which question the reliability and validity of the published results, the *PLOS One* Editors agreed to retract the article.

All authors agree with the retraction and apologize for the issues with the published article.
